# Integrative analysis identifies potential ferroptosis-related genes of hypoxia adaptation in yak

**DOI:** 10.3389/fvets.2022.1022972

**Published:** 2022-10-11

**Authors:** Jian Zhang, Yan Cui

**Affiliations:** ^1^College of Veterinary Medicine, Gansu Agricultural University, Lanzhou, China; ^2^Technology and Research Center of Gansu Province for Embryonic Engineering of Bovine and Sheep & Goat, Lanzhou, China

**Keywords:** yak, oviduct, hypoxia adaptation, ferroptosis, bulk transcriptomics

## Abstract

There are studies on the hypoxia adaptation in yak, but there are few studies on the regulation of ferroptosis by hypoxia. This study was the first time to explore ferroptosis-related genes about hypoxia in yak. In this study, the oviduct epithelial cells between yak and bovine are performed by integrative analysis for functions, regulating network and hub genes. The results showed 29 up-regulated ferroptosis genes and 67 down-regulated ferroptosis genes, and GO-KEGG analysis showed that up-regulated differentially expressed genes (DEGs) were significantly enriched in ribosome pathway and oxidative phosphorylation pathway. Down-regulated DEGs were significantly enriched in longevity regulating pathway-mammal pathway. Mitophagy-Animal Pathway was a significant enrichment pathway for the up-regulated differentially expressed ferroptosis genes (DE-FRGs). HIF-1 signaling pathway is a significant pathway for the down-regulated DE-FRGs. By constructing DE-FRGs protein-protein interaction (PPI) network, 10 hub DE-FRGs (Jun, STAT3, SP1, HIF1A, Mapk1, Mapk3, Rela, Ulk1, CDKN1A, EPAS1) were obtained. The bta-mir-21-5p, bta-mir-10a and bta-mir-17-5p related to STAT3 were predicted. The results of this study indicated the important genes and pathways of the hypoxia in yak, and it was the first time to study ferroptosis genes and pathways related to the hypoxia adaptation by bulk-seq in yak. This study provided sufficient transcriptome datas for hypoxia adaptation.

## Introduction

Yaks are a hypoxia-tolerant species that live in a high-altitude environment (1), which can adapt to hypoxia and cold environment (2). Studies have been carried out on hypoxia adaptation of Yak Gluteus (3), heart tissues (4), and lung (5), but there were few studies carried out on the hypoxia adaptation in yak oviduct, which is an important organ for transporting sperm, oocytes and oosperm, and it is the first environment for early embryo exposure. Therefore, this study focuses on the hypoxia adaptation of oviduct.

Ferroptosis is a popular research topic, which is a new type of iron-dependent programmed cell death, which is different from apoptosis, cell necrosis and autophagy. Ferroptosis leads to smaller mitochondria, increased membrane density, decreased cristae, and no obvious morphological changes in nucleus. Hypoxia inhibits ferritinophagy, increases in mitochondrial ferritin, and protects from ferroptosis (6). Hypoxia regulates the expression of ferroptosis genes in acute myocardial infarction (7), osteoclasts (8), gastric cancer (9), and hepatocellular Carcinoma (10). The above research indicates that hypoxia has a regulatory effect on ferroptosis. Yak is a plateau animal living in low oxygen environment, but there is no research on ferroptosis in yak. The mechanism of ferroptosis in hypoxia adaptation remains unclear. In addition, many ferroptosis genes have not been discovered, so it is necessary to further study ferroptosis genes.

At present, we used Weighted Correlation Network Analysis (WGCNA) to identify hub genes of hypoxic adaptation on lung, muscle, and spleen (11), RNA-Seq analysis also was used to analysis yak ovary (12). But there is no bioinformatics research on the mechanism of ferroptosis genes in hypoxia adaptation. As a result, we used data mining and data analysis techniques to screen DEGs between yak and bovine, and DE-FRGs were obtained from the DEGs and ferroptosis data. DE-FRGs were enriched in functional pathways and analyzed to construct a protein-protein interaction (PPI) network. Then, we screened for ferroptosis-related hub genes. Our results will contribute to the understanding of ferroptosis genes and provide new research thought. They were also basic datas for the study of hypoxia adaptation.

## Materials and methods

### Sample collection and microarray data collection

Yak oviduct samples were collected from Xining city, Qinghai province, China, at an altitude of 3,800~4,500 m. We performed the sequencing analysis, storing dataset in the Genome Sequence Archive (https://ngdc.cncb.ac.cn/gsa/) with series Numbers CRA007411. We selected a dataset of isthmus epithelial cells and ampulla epithelial cells of the oviduct. The RNA expression data of bovine were downloaded from the GEO (http://www.ncbi.nlm.nih.gov/geo/) database with series numbers GSE124110 (13). Apart from that, Munich has an altitude of about 520 m. 259 ferroptosis-related genes were retrieved from the public FerrDb database (Table 1) (http://www.zhounan.org/ferrdb/current/).

**Table 1:** Two hundred fifty-nine ferroptosis-related genes.

RPL8, IREB2, ATP5MC3, CS, EMC2, ACSF2, NOX1, CYBB, NOX3, NOX4, NOX5, DUOX1, DUOX2, G6PD, PGD, VDAC2, PIK3CA, FLT3, SCP2, TP53, ACSL4, LPCAT3, NRAS, KRAS, HRAS, TF, TFRC, TFR2, SLC38A1, SLC1A5, GLS2, GOT1, CARS1, ALOX5, KEAP1, HMOX1, ATG5, ATG7, NCOA4, ALOX12, ALOX12B, ALOX15, ALOX15B, ALOXE3, PHKG2, ACO1, G6PDX, ULK1, ATG3, ATG4D, BECN1, MAP1LC3A, GABARAPL2, GABARAPL1, ATG16L1, WIPI1, WIPI2, SNX4, ATG13, ULK2, SAT1, EGFR, MAPK3, MAPK1, BID, ZEB1, DPP4, CDKN2A, PEBP1, SOCS1, CDO1, MYB, MAPK8, MAPK9, CHAC1, MAPK14, LINC00472, PRKAA2, PRKAA1, ELAVL1, BAP1, BCC1, MIR6852, ACVR1B, TGFBR1, EPAS1, HILPDA, HIF1A, IFNG, ANO6, LPIN1, HMGB1, TNFAIP3, TLR4, ATF3, ATM, YY1AP1, EGLN2, MIOX, TAZ, MTDH, IDH1, SIRT1, FBXW7, PANX1, DNAJB6, BACH1, LONP1, PTGS2, DUSP1, NOS2, NCF2, MT3, UBC, ALB, TXNRD1, SRXN1, GPX2, BNIP3, OXSR1, SELENOS, ANGPTL7, SLC7A11, DDIT4, LOC284561, ASNS, TSC22D3, DDIT3, JDP2, SESN2, SLC1A4, PCK2, TXNIP, VLDLR, GPT2, PSAT1, LURAP1L, SLC7A5, HERPUD1, XBP1, SLC3A2, CBS, ATF4, ZNF419, KLHL24, TRIB3, ZFP69B, ATP6V1G2, VEGFA, GDF15, TUBE1, ARRDC3, CEBPG, SNORA16A, RGS4, BLOC1S5-TXNDC5, LOC390705, EIF2S1, KIM-1, IL6, CXCL2, RELA, HSD17B11, AGPAT3, SETD1B, FTL, MAFG, IL33, FTH1, SLC40A1, GPX4, HAMP, HSPB1, NFE2L2, STEAP3, DRD5, DRD4, MAP3K5, SLC2A1, SLC2A3, SLC2A6, SLC2A8, SLC2A12, GLUT13, SLC2A14, EIF2AK4, TFAP2C, SP1, HBA1, NNMT, PLIN4, HIC1, STMN1, RRM2, CAPG, HNF4A, NGB, YWHAE, GABPB1, AURKA, MIR4715, RIPK1, PRDX1, MIR30B, AKR1C1, AKR1C2, AKR1C3, RB1, HSF1, GCLC, SQSTM1, NQO1, MUC1, MT1G, CISD1, FANCD2, FTMT, HSPA5, HELLS, SCD, FADS2, SRC, STAT3, PML, MTOR, NFS1, TP63, CDKN1A, MIR137, ENPP2, FH, CISD2, MIR9-1, MIR9-2, MIR9-3, ISCU, ACSL3, OTUB1, CD44, LINC00336, BRD4, PRDX6, MIR17, NF2, ARNTL, JUN, CA9, TMBIM4, PLIN2, MIR212, Fer1HCH, AIFM2, LAMP2, ZFP36, PROM2, CHMP5, CHMP6, CAV1, GCH1.

### Identification of DEGs and DE-FRGs

We compared the data sets of yak and bovine, selection criteria for adjusted-*P* < 0.05 and |log_2_FC| > 1. Under these screening conditions, some DEGs were identified, and volcano plot and heatmap showed a better repeatability of data. Then we devided DEGs into up-DEGs and down-DEGs, then these differential genes and ferroptosis genes were analyzed by Venn website (http://bioinformatics.psb.ugent.be/webtools/Venn/). These DE-FRGs were used for subsequent analysis.

### Functional enrichment analyses for DEGs and DE-FRGs

The above DEGs and DE-FRGs were extracted, and then they were used for GO (Gene Ontology, GO) functional enrichment and KEGG (Kyoto Encyclopedia of Genes and Genomes, KEGG) signal pathway enrichment analysis for reference genome Bos grunniens by DAVID online database (https://David.ncifcrf.gov/). GO contains molecular functions (MF), biological processes (BP), and cellular component (CC). KEGG database includes biological functions, diseases, chemicals and drugs. Adjusted *P*-value < 0.05 were defined as statistically significant.

### Construction of PPI of DE-FRGs

To further explore differential protein-protein interactions, protein interaction networks were analyzed for DE-FRGs using online String 11.5 database (https://cn.string-db.org/). The obtained results were imported into Cytoscape software for visualization and a confidence score > 0.40 was set to screen PPI pairs and build a PPI network.

### Identification of hub genes and miRNAs

In protein network regulation results, nodes represent proteins and lines represent interactions between proteins. The hub genes were screened and scored by Cytohubba 3.7.1 plug-in in Cytoscape software. NetworkAnalyst (https://www.networkanalyst.ca/NetworkAnalyst/home.xhtml), an online software, which can help people find miRNA-interactions in gene regulation networks. Using B. taurus (cow) as ID type, 10 hub genes were input to search for miRNA related to hub genes.

## Results

### Differential expression analysis of DEGs and DE-FRGs

**Figure 1:**
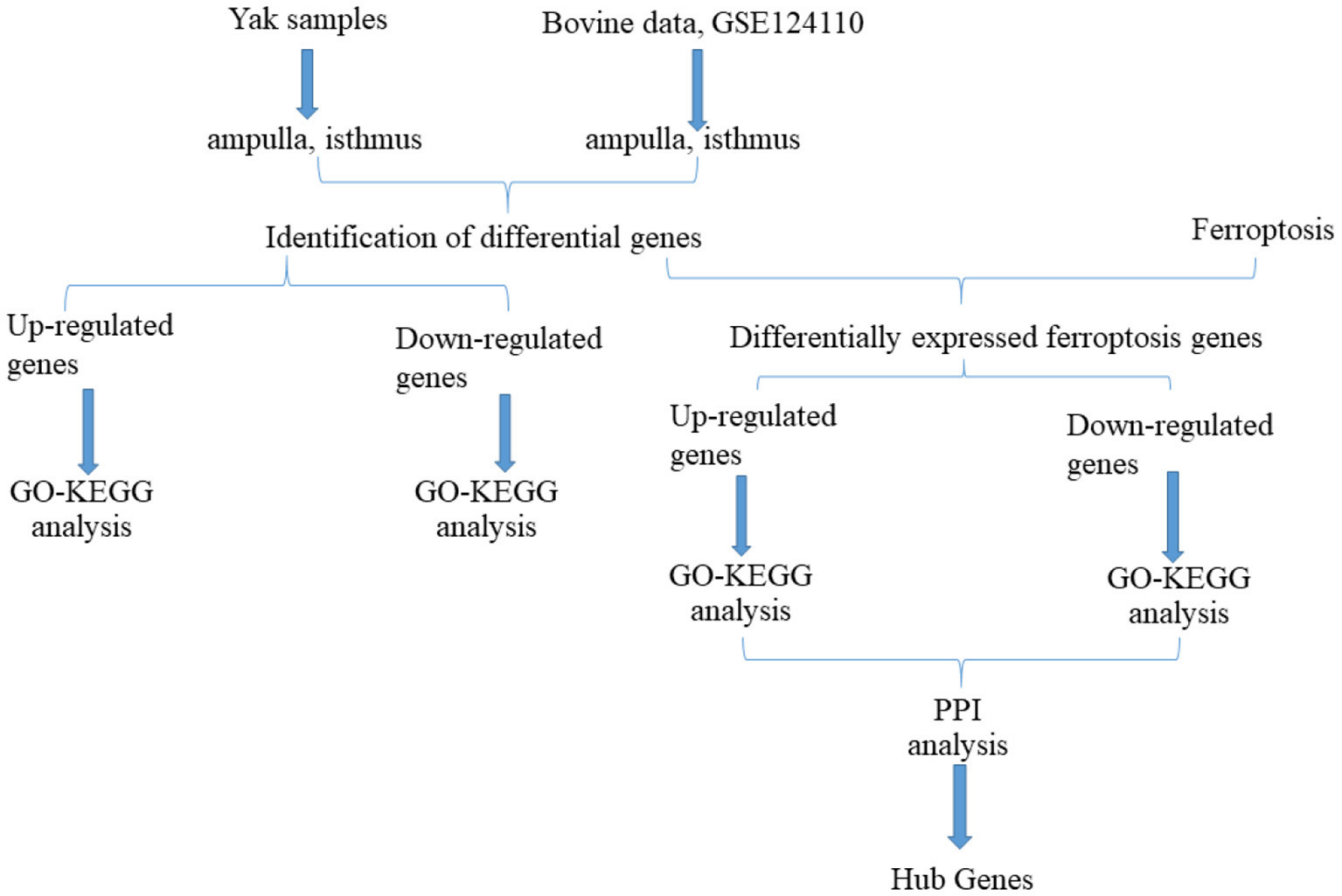
Flow chart of this study.

**Figure 2:**
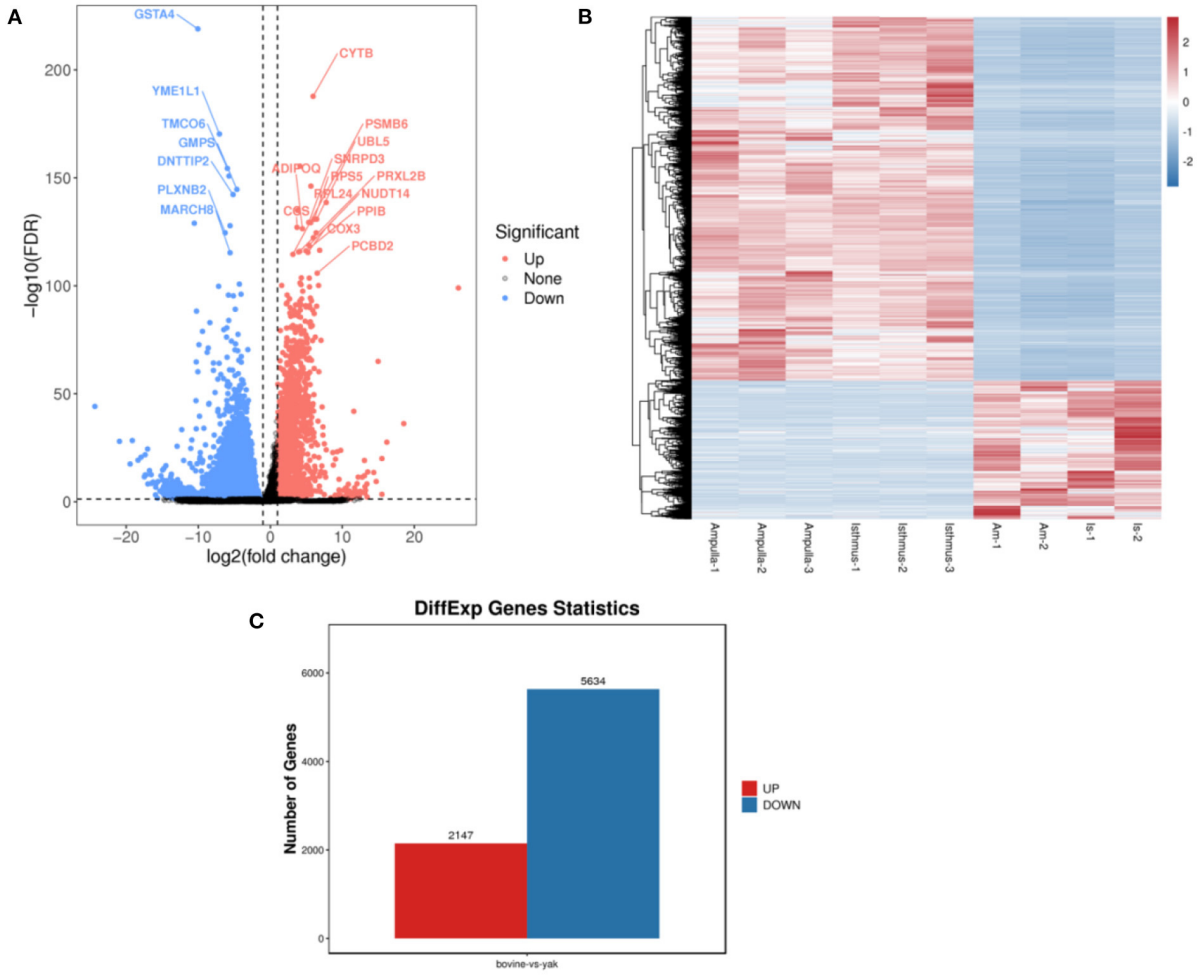
Comparison of yak and bovine oviduct epithelial cells. **(A)** The volcano plot of DEGs. **(B)** The heatmap of DEGs. **(C)** The number of up-regulated DEGs and down-regulated DEGs.

**Figure 3:**
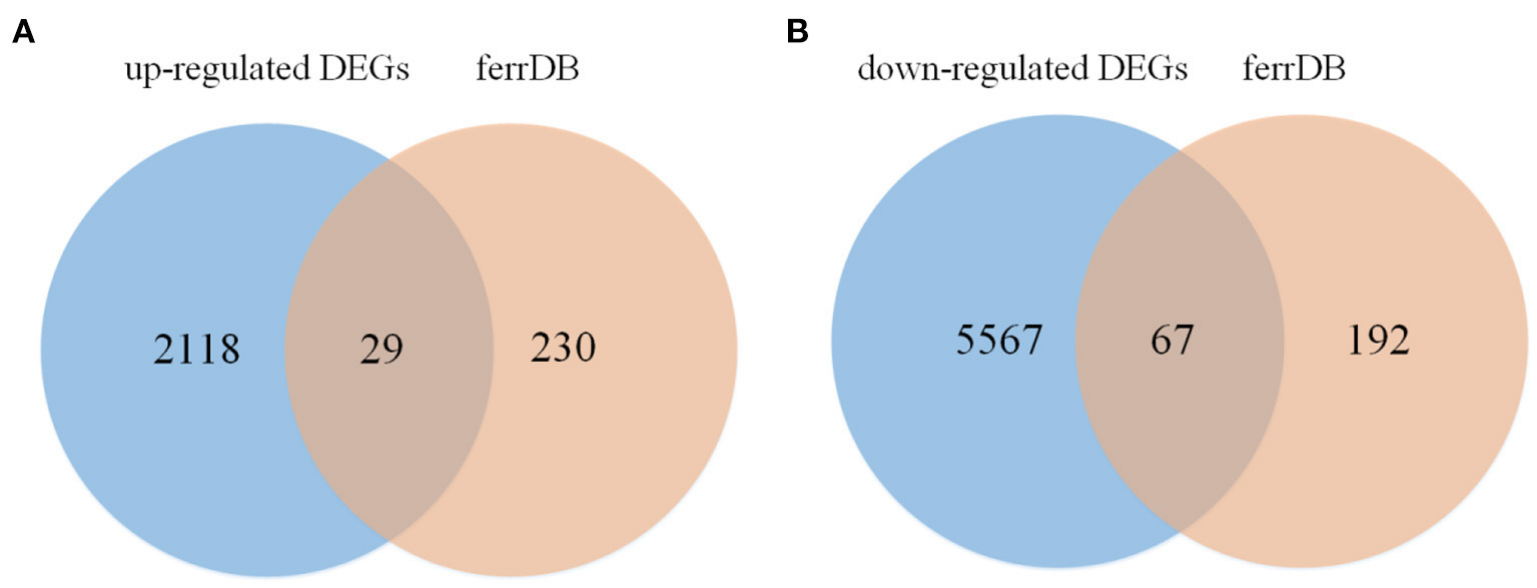
Venn diagram displaying the DE-FRGs. **(A)** The venn of up-regulated DE-FRGs. **(B)** The venn of down-regulated DE-FRGs.

The flow chart of this study is shown in Figure 1. All repeatability of used data sets were with good biological significance by volcano plot (Figure 2A) and heatmap (Figure 2B). We found that CYTB and PSMB6 were significantly up-regulated genes, while GSTA4, YME1L1, and TMCO6 were significantly down-regulated genes. Different genes were obtained by comparing yak and bovine, including 2,147 up-regulated differential genes and 5,634 down-regulated differential genes (Figure 2C). We intersected these differential genes with ferroptosis genes, respectively, and found 67 down-regulated ferroptosis genes (Figure 3A) and 29 up-regulated ferroptosis genes (Figure 3B). We present all DE-FRGs (Table 2).

**Table 2:** Differentially expressed ferroptosis genes.

**Up-regulated ferroptosis genes**	**Down-regulated ferroptosis genes**
ATP5MC3, KRAS, HRAS, TF, MAP1LC3A, GABARAPL2, SAT1, CHAC1, IFNG, TNFAIP3, ATF3, TAZ, DNAJB6, MT3, SELENOS, DDIT3, HERPUD1, ATF4, HSPB1, CAPG, CISD1, HSPA5, CDKN1A, OTUB1, PRDX6, JUN, AIFM2, CHMP6, GCH1.	IREB2, G6PD, PIK3CA, NRAS, TFRC, SLC1A5, GLS2, GOT1, NCOA4, ALOX15, ULK1, ATG4D, ATG16L1, ATG13, MAPK3, MAPK1, MYB, PRKAA2, PRKAA1, ELAVL1, ABCC1, ACVR1B, TGFBR1, EPAS1, HIF1A, ANO6, LPIN1, TLR4, IDH1, PANX1, BACH1, LONP1, DUSP1, NOS2, VLDLR, GPT2, PSAT1, LURAP1L, SLC7A5, XBP1, KLHL24, ARRDC3, RELA, SETD1B, NFE2L2, STEAP3, MAP3K5, SLC2A1, SLC2A12, EIF2AK4, SP1, GABPB1, RIPK1, RB1, HSF1, SQSTM1, NQO1, SCD, SRC, STAT3, PML, MTOR, ENPP2, NF2, CA9, LAMP2, PROM2.

### Functional annotation of DEGs

**Figure 4:**
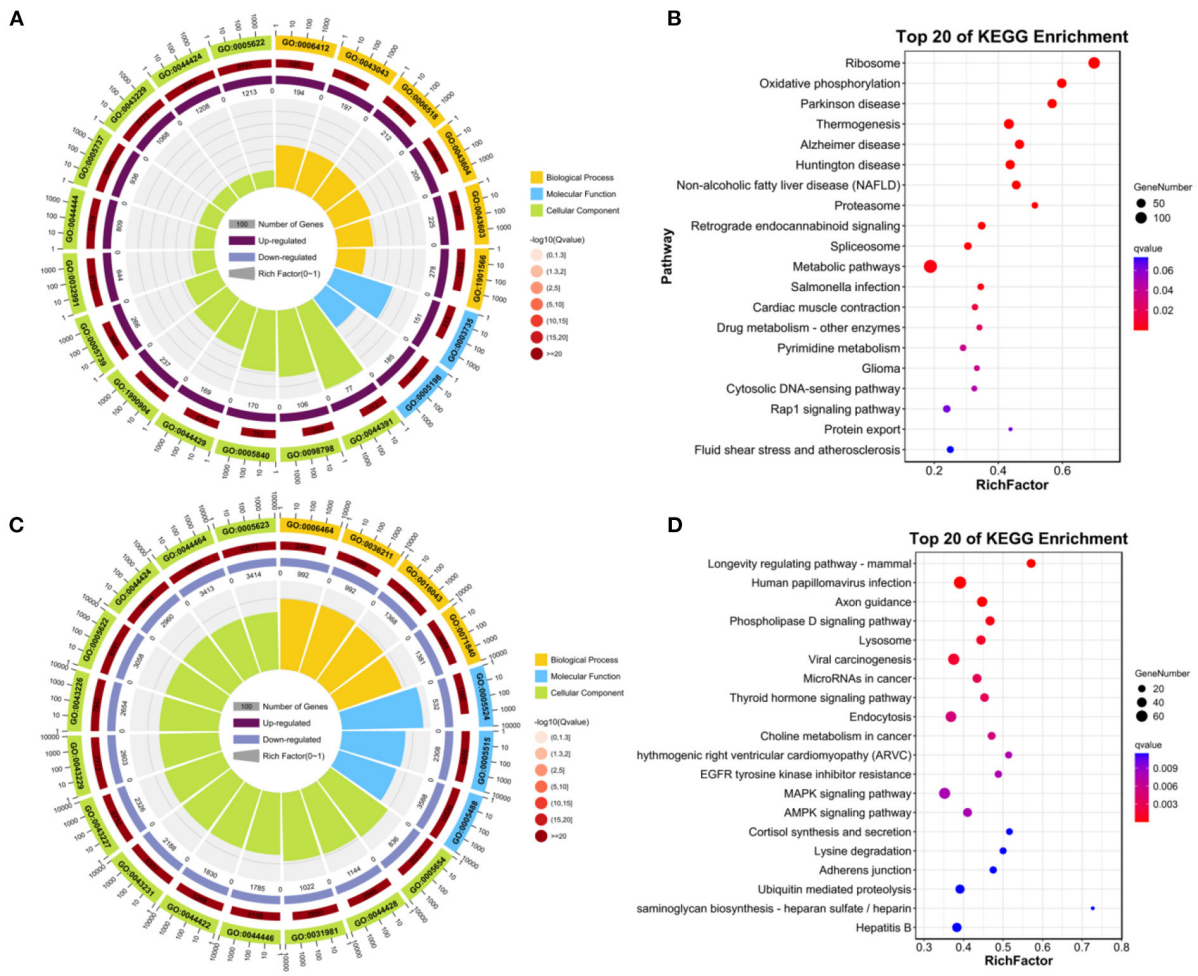
Enrichment analysis of DEGs. **(A)** GO enrichment analysis of up-regulated DEGs. **(B)** KEGG analysis of up-regulated DEGs. **(C)** GO enrichment analysis of down-regulated DEGs. **(D)** KEGG analysis of down-regulated DEGs.

Functional enrichment and KEGG pathway analysis were performed on 2,147 up-DEGs. As shown in Figure 4A (*p*-value < 0.05), changes in GO biological processes (BP) mainly included cellular process, metabolic process, biological regulation, regulation of biological process, response to stimulus, cellular component organization or biogenesis, localization, positive regulation of biological process, multicellular organismal process, negative regulation of biological process, signaling, developmental process, immune system process. Changes in cellular component (CC) mainly focus on cell, cell part, organelle, organelle part, protein-containing complex, membrane, membrane-enclosed lumen, membrane part, extracellular region. The molecular function (MF) includes binding, catalytic activity, structural molecule activity, molecular function regulator, transporter activity, transcription regulator activity, molecular transducer activity. In particular (Figure 4B), ribosome and oxidative phosphorylation were two pathways with abundant concentration and significant differences. Thermogenesis pathway, Alzheimer's disease pathway, huntington disease pathway, NAFLD pathway were another pathway with significant differences.

Functional enrichment and KEGG pathway analysis were performed on 5,634 down-DEGs. As shown in Figure 4C (*p*-value < 0.05), changes in GO biological processes (BP) mainly included cellular process, biological regulation, metabolic process, regulation of biological process, response to stimulus, cellular component organization or biogenesis, localization, multicellular organismal process, positive regulation of biological process, signaling, developmental process, negative regulation of biological process. Changes in cellular component (CC) mainly focus on cell, cell part, organelle, organelle part, membrane, protein-containing complex, membrane part. In addition, in the molecular function (MF), the main changes are binding, catalytic activity, transcription regulator activity, transporter activity, molecular function regulator, membrane-enclosed lumen. KEGG pathway analysis was rich in longevity regulating pathway-mammal pathway, human papillomavirus infection, axon guidance, phospholipase D signaling pathway, Lysosome, viral carcinogenesis, thyroid hormone signaling pathway (Figure 4D).

### Functional annotation of DE-FRGs

**Figure 5:**
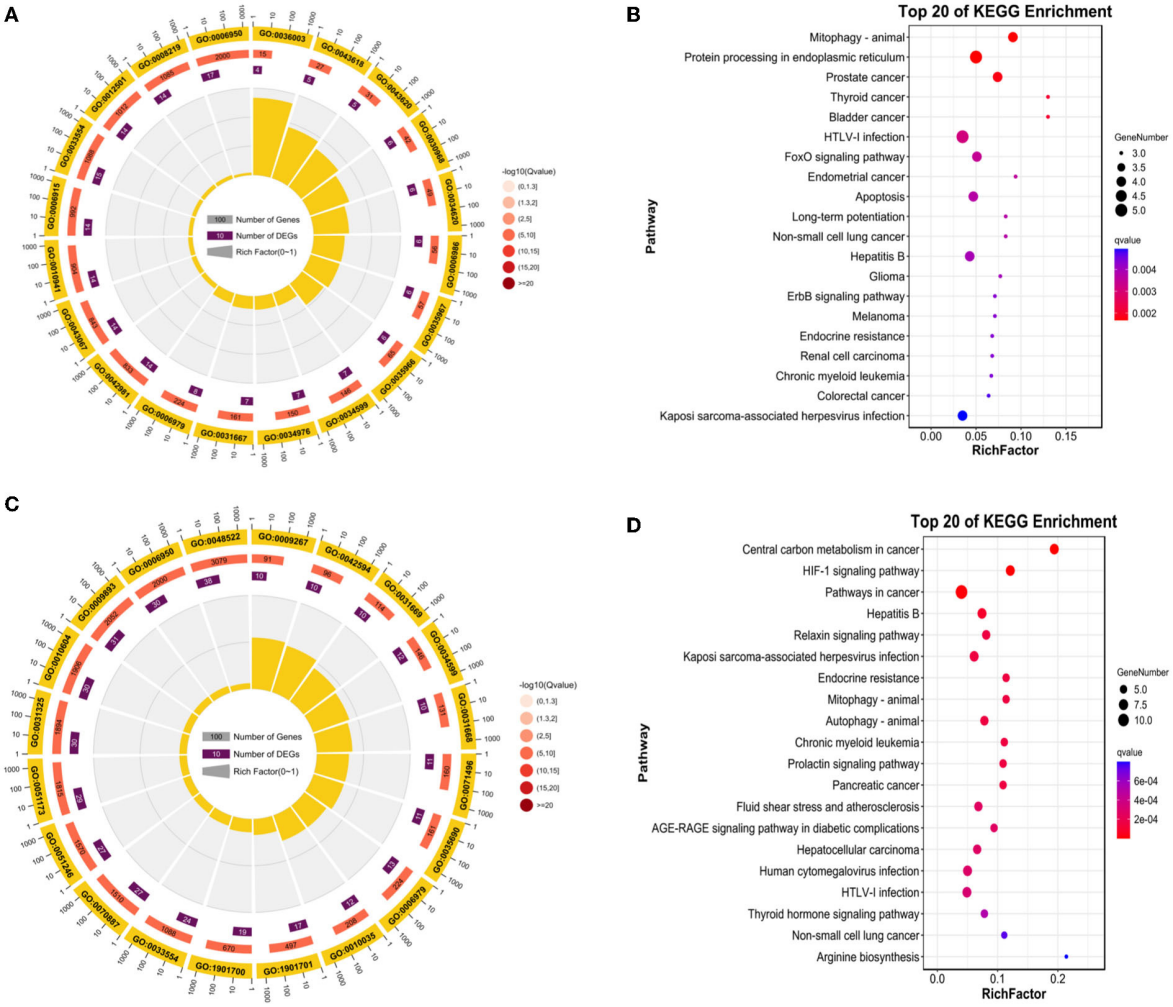
Enrichment analysis of DE-FRGs. **(A)** GO enrichment analysis of up-regulated DE-FRGs. **(B)** KEGG analysis of up-regulated DE-FRGs. **(C)** GO analysis of down-regulated DE-FRGs. **(D)** KEGG enrichment analysis of down-regulated DE-FRGs.

As shown in Figure 5A (*p*-value < 0.05), 29 up-regulated DE-FRGs changes in GO biological processes (BP) mainly included cellular process, metabolic process, biological regulation, regulation of biological process, response to stimulus, negative regulation of biological process, positive regulation of biological process, cellular component organization or biogenesis, signaling, localization, developmental process, multicellular organismal process, multi-organism process. Changes in cellular component (CC) mainly focus on cell, cell part, organelle, organelle part, membrane, protein-containing complex, membrane-enclosed lumen, membrane part, In addition, In the molecular function (MF), binding, catalytic activity, molecular function regulator, transcription regulator activity. KEGG pathway analysis is rich in mitophagy-animal pathway, protein processing in endoplasmic reticulum pathway, prostate cancer pathway (Figure 5B).

As shown in Figure 5C (*p*-value < 0.05), 67 down-regulated DE-FRGs changes in GO biological processes (BP) mainly included cellular process, biological regulation, metabolic process, regulation of biological process, response to stimulus, localization, positive regulation of biological process, cellular component organization or biogenesis, negative regulation of biological process, signaling, multicellular organismal process, developmental process, immune system process, multi-organism process. Changes in cellular component (CC) mainly focus on cell, cell part, organelle, membrane, organelle part, protein-containing complex, membrane part, membrane-enclosed lumen. In the molecular function (MF), binding, catalytic activity, transcription regulator activity, transporter activity, In particular, KEGG pathway analysis is rich in central carbon metabolism in cancer pathway, HIF-1 signaling pathway, pathways in cancer, hepatitis B pathway, relaxin signaling pathway, Kaposi sarcoma-associated herpesvirus infection pathway (Figure 5D).

### PPI network analysis of DE-FRGs

**Figure 6:**
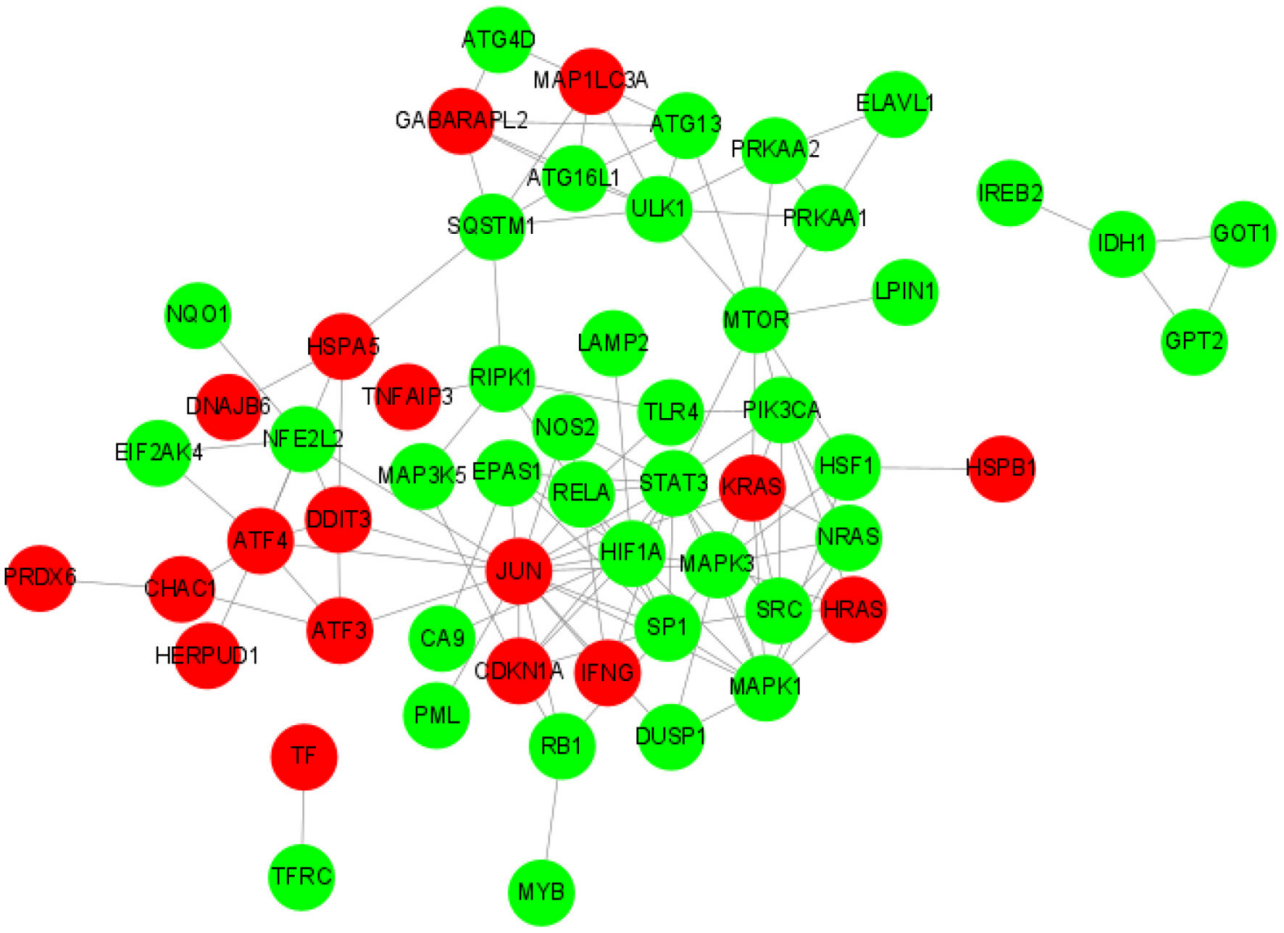
PPI network of DE-FRGs.

String software was used to analyze (https://cn.string-db.org/). We made the protein-protein interaction network (Figure 6). Differential genes in network string interactions short.tsv ranked by cytoHubba method. The up-regulated DE-FRGs were: AIFM2, ATF3, ATF4, ATP5MC3, CAPG, CDKN1A, CHAC1, CHMP6, CISD1, DDIT3, DNAJB6, GABARAPL2, GCH1, HERPUD1, HRAS, HSPA5, HSPB1, IFNG, JUN, KRAS, MAP1LC3A, MT3, OTUB1, PRDX6, SAT1, SELENOS, TAZ, TF, and TNFAIP3. The down-regulated DE-FRGs were: ABCC1, ACVR1B, ALOX15, ANO6, ARRDC3, ATG13, ATG16L1, ATG4D, BACH1, CA9, DUSP1, EIF2AK4, ELAVL1, ENPP2, EPAS1, G6PD, GABPB1, GLS2, GOT1, GPT2, HIF1A, HSF1, IDH1, IREB2, KLHL24, LAMP2, LONP1, LPIN1, LURAP1L, MAP3K5, MAPK1, MAPK3, MTOR, MYB, NCOA4, NF2, NFE2L2, NOS2, NQO1, NRAS, PANX1, PIK3CA, PML, PRKAA1, PRKAA2, PROM2, PSAT1, RB1, RELA, RIPK1, SCD, SETD1B, SLC1A5, SLC2A1, SLC2A12, SLC7A5, SP1, 3SQSTM1, SRC, STAT3, STEAP3, TFRC, TGFBR1, TLR4, ULK1, VLDLR, and XBP1. JUN regulates RELA, MAPK3, NOS2, STAT3, NFE2L2, KRAS, SP1, RB1, PML, and MAPK1. KRAS regulates NRAS, PIK3CA, MAPK3, SRC, MTOR, and MAPK1. CDKN1A regulates SP1, HIF1A, JUN, RB1, MAP3K5, and STAT3. DDIT3 regulates HSPA5, NFE2L2, and JUN.

### Hub DE-FRGs

**Figure 7:**
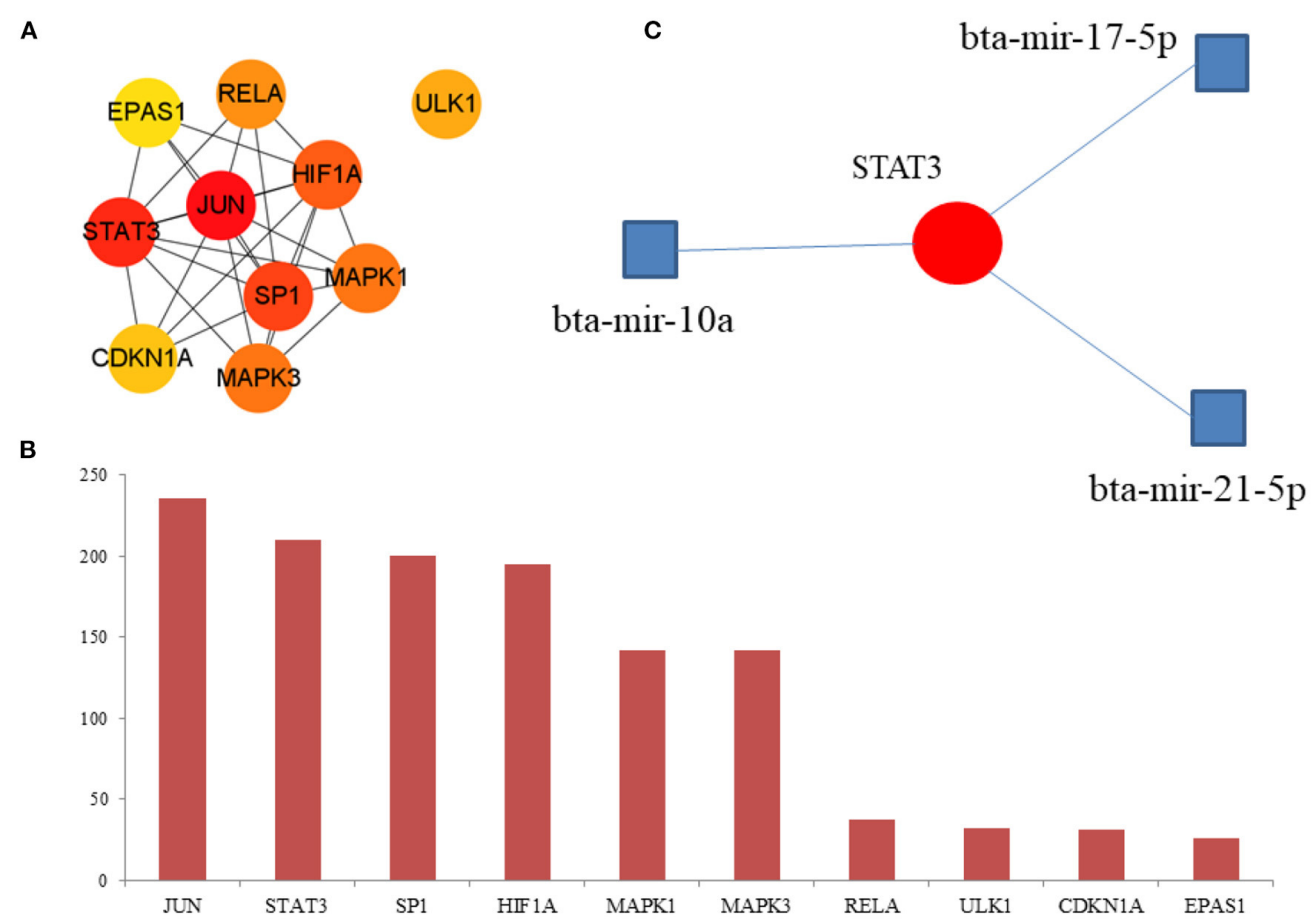
Hub DE-FRGs and miRNA. **(A)** Network of the top 10 hub DE-FRGs. **(B)** The rank of the hub DE-FRGs. **(C)** Prediction of the miRNA.

The hub DE-FRGs were ranked as follows (Figures 7A,B): JUN, STAT3, SP1, HIF1A, MAPK1, MAPK3, RELA, ULK1, CDKN1A, and EPAS1. JUN and CDKN1A are up-regulated DE-FRGs. STAT3, SP1, HIF1A, MAPK1, MAPK3, RELA, EPAS1, and ULK1 are down-regulated DE-FRGs. Among them, JUN, STAT3, SP1, and HIF1A are the most significant hub genes of hypoxia adaptation involved in ferroptosis. We used NetworkAnalyst (https://www.networkanalyst.ca/NetworkAnalyst/home.xhtml) for the first 10 hub genes for relevant miRNA prediction. Then, bta-mir-21-5p, bta-mir-10a and bta-mir-17-5p associated with STAT3 (Figure 7C). However, these findings require further exploration.

## Discussion

By comparing the oviduct epithelial cells of yak and bovine, the key genes and pathways of hypoxia adaptation were identified, and the key genes related to ferroptosis in hypoxia adaptation of yak were further discussed. We found that CYTB, PSMB6, GSTA4, YME1L1, and TMCO6 are the key genes of hypoxia adaptation in yak, and CYTB is also the key gene of hypoxia adaptation in previous studies (14). In the study of the hypoxic microenvironment of cancer, mitochondrial DNA-encoded Cytb was ~30% lower in Lewis lung carcinoma hearts (15), heteroplasmic changes were found in ND1 and CYTB in epithelioma glandulae sebacei and in CYTB in lymphoma centroblasticum (16). All the above studies on CYTB in hypoxia indicate that hypoxia regulates the expression of CYTB, which is consistent that CYTB is a key gene for hypoxia adaptation in yaks. In this study, the ribosome pathway and oxidative phoenix pathway are the key pathways of hypoxia adaptation. Oxidative phoenix pathway leads to the production of ATP in biological oxidation, and yak needs to consume more ATP in hypoxia environment, which may be the key pathway of oxidative phoenix pathway for adaptation to high altitude. The results of this study have been replicated in other mammals, hypoxia induced the expression of oxidative phoenix pathway in mammals (17) and an Asian pika (18), which was consistent with hypoxia adaptation of yak.

Ferroptosis is a unique cell death mode driven by iron-dependent phospholipid peroxidation, which is regulated by a variety of cellular metabolic pathways, including REDOX homeostasis, iron metabolism, mitochondrial activity and metabolism of amino acids, lipids and sugars. Cellular iron is essential for maintaining multiple metabolic pathways, and excess free iron may cause oxidative damage or provoke cell death. Hypoxic primary human macrophages have reduced free iron and increased expression of ferritin, including mitochondrial ferritin (FTMT), to store iron. The relationship between hypoxia and iron death has attracted the attention of scholars. There is no high-throughput study to explore the potential link between ferroptosis and hypoxia in yak, and the mechanism of ferroptosis in hypoxic adaptation. We obtained 96 DE-FRGs by intersecting the differential genes of hypoxia adaptation with FerrDb, including 29 up-regulated DE-FRGs and 67 down-regulated DE-FRGs. Then, the enrichment analysis of DE-FRGs with KEGG-GO showed that the up-regulated ferroptosis genes were mainly involved in Mitophagy-animal pathway, while the down-regulated ferroptosis genes were mainly involved in HIF-1 signaling pathway, which indicated that hypoxia might inhibit the oxygen metabolism pathway related to ferroptosis. Mitophagy-biological pathway is the enrichment pathway of DE-FRGs. In previous studies, hypoxia can activate the PINK1/Parkin-mediated mitophagy pathway (19), selective activation of mitophagy might promote cell survival under hypoxic conditions (20), breast cancer (21), and pulmonary fibrosis (22). Tumor cells were exposed to hypoxia, the yak was in the hypoxic environment, the down-regulated DE-FRGs in the oviduct is also enriched in HIF-1 signaling pathway, HIF-1 is a central regulator of cellular adaptation to hypoxia (23), positively selected hypoxia-related genes in the buff-throated partridge were distributed in the HIF-1 signaling pathway (24), Hypoxia Enhances HIF-1α Transcription Activity by Upregulating KDM4A and Mediating H3K9me3 (25), suppression of the HIF-1 signaling pathway by microRNA regulation may play a key role in the pathogenesis of un-acclimatization with high altitude hypoxia (26), HIF-1 signaling pathway is an important topic in high-altitude medicine (27), the previous hypoxia research were consistent with this study. Therefore, HIF-1 signaling pathway may play an important role in the regulation of ferroptosis by hypoxia-induced. This study analyzed the hypoxia adaptation from the perspective of bioinformatics, and further analyzed the ferroptosis genes related to the hypoxia adaptation, which can provide an effective reference for the study of hypoxia adaptation in yak.

Finally, we analyzed the hub DE-FRGs, and found that JUN, STAT3, SP1 and HIF1A are the most significant hub DE-FRGs. JUN is also the hub genes of ferroptosis-related genes of Alzheimer's disease (28). STAT3-induced lysosomal membrane permeabilization (29). HIF-1 is ubiquitous in human and mammalian cells, and also expressed under normal oxygen (21% O_2_), but the synthesized HIF-1 protein degrades quickly in cells, and HIF-1 can be stably expressed only under hypoxia. The HIF target MAFF promotes tumor invasion and metastasis through IL11 and STAT3 signaling (30), HIF-1α facilitates osteocyte-mediated osteoclastogenesis by activating JAK2/STAT3 pathway (31), which indicated that HIF regulated the expression of STAT3, but the regulation of HIF on STAT3 should be further studied, which is related to hypoxia adaptation in yak.

In this study, the hypoxia adaptation of yak oviduct was studied, and the genes regulating hypoxia adaptation were excavated CYTB, PSMB6, GSTA4, YME1L1 and TMCO6. These new genes provide new research directions. We found that Mitophagy-animal pathway and HIF-1 signaling pathway were very important for the study of ferroptosis genes regulated by hypoxia. In addition, miRNA related to STAT3 is also a potential biomolecule for the following study of hypoxia. In the future, hypoxia adaptation can be explored from the perspective of miRNA. The mining of these genes and pathways provides new targeted molecules for the study of high altitude adaptation, new omics data for the study of hypoxia-regulated ferroptosis genes, which are also basic data for the study of hypoxia adaptation.

## Data availability statement

The authors acknowledge that the transcriptome data stored in a database of GSA (https://ngdc.cncb.ac.cn/gsa/), the data under accession CRA007411 are publicly available.

## Ethics statement

The animal study was reviewed and approved by the Animal Research Ethics Committee of Gansu Agricultural University. Written informed consent was obtained from the owners for the participation of their animals in this study.

## Author contributions

JZ were responsible for the concept and design of this study. YC processed the data. All authors listed have made a substantial, direct, and intellectual contribution to the work and approved it for publication.

## Funding

This study was supported by the Nature Science Foundation of China (Grant No. 31972634).

## Conflict of interest

The authors declare that the research was conducted in the absence of any commercial or financial relationships that could be construed as a potential conflict of interest.

## Publisher's note

All claims expressed in this article are solely those of the authors and do not necessarily represent those of their affiliated organizations, or those of the publisher, the editors and the reviewers. Any product that may be evaluated in this article, or claim that may be made by its manufacturer, is not guaranteed or endorsed by the publisher.
